# Impact of IL-8 on survival after TARE in HCC: a comprehensive investigation and external validation from the SORAMIC trial

**DOI:** 10.1007/s00432-024-05947-4

**Published:** 2024-11-06

**Authors:** Aaron Schindler, Janett Fischer, Anne-Bettina Beeskow, Thomas Lincke, Sebastian Ebel, Daniel Seehofer, Timm Denecke, Rhea Veelken, Osama Sabri, Osman Öcal, Max Seidensticker, Thomas Berg, Florian van Bömmel

**Affiliations:** 1 Division of Hepatology, Department of Medicine II, Leipzig University Medical Center, Liebigstrasse 20, 04103 Leipzig, Germany; 2 Department of Diagnostic and Interventional Radiology, Leipzig University Medical Center, Leipzig, Germany; 3 Department of Nuclear Medicine, Leipzig University Medical Center, 04103 Leipzig, Germany; 4 Department of Visceral, Thoracic and Vascular Surgery, Leipzig University Medical Center, Leipzig, Germany; 5 University Liver Tumor Center (ULTC), Leipzig University Medical Center, Leipzig, Germany; 6 Division of Hepatology, Department of Medicine II, Laboratory for Clinical and Experimental Hepatology, Leipzig University Medical Center, Leipzig, Germany; 7 Department of Radiology, University Hospital, LMU Munich, Munich, Germany

**Keywords:** Biomarkers, Liver cancer, Translational research, Radiotherapy

## Abstract

**Purpose:**

In the treatment of hepatocellular carcinoma (HCC) with transarterial radioembolization (TARE), identifying reliable biomarkers for predicting survival outcomes remains a critical challenge. We aimed to address this gap by investigating the significance of serum cytokines associated with inflammation as potential biomarkers for the selection of patients for TARE.

**Methods:**

Our retrospective study involved 161 patients diagnosed with HCC who underwent Y90 radioembolization at our medical center between 2010 and 2020. Serum samples from a subset of 78 patients were retrospectively analyzed to determine the concentrations of pro-inflammatory cytokines. The results from the prospective SORAMIC trial were used for independent validation.

**Results:**

With a median overall survival of 36 weeks (range 4-436), our study showed the strongest correlation between 12-week survival and IL-8 levels before treatment (*p* < 0.001), while other relevant interleukins, interferon-α2, INF-γ, TNF-α and MCP-1 were not associated with survival. IL-8 levels below the cut-off of 190 pg/mL were significantly associated with increased 12-week and 24-week survival, with hazard ratios of 19.01 (95% CI: 2.29-157.89) and 2.57 (95% CI: 1.05–6.31), respectively (*p* = 0.006 and *p* = 0.039, respectively). In the adjusted multivariate analysis, the 190 pg/mL cut-off for IL-8 remained independently associated with 12- (*p* = 0.011) and 24-week survival (*p* = 0.039). Similarly, the SORAMIC population showed a strong association between IL-8 levels and 36-week survival (*p* = 0.03).

**Conclusion:**

Our study emphasizes the pivotal role of IL-8 as a valuable parameter, demonstrating its potential for predicting treatment outcomes and assessing liver function in patients with HCC undergoing TARE. The robustness of these findings warrants further validation.

**Supplementary Information:**

The online version contains supplementary material available at 10.1007/s00432-024-05947-4.

## Introduction

Hepatocellular carcinoma (HCC) is the most common type of primary liver cancer and the third leading cause of cancer-related deaths worldwide (Forner et al. 2018; WHO 2020). Treatment options for HCCs depend on the stage of the disease and may include surgery, locoregional and systemic treatments (EASL 2018, Vogel et al. 2021). The Barcelona Clinic Liver Cancer (BCLC) staging system is widely used to classify and stage HCCs, considering various factors including tumor characteristics, liver function, and the patient’s general health (Reig et al. 2021). The BCLC system also provides treatment recommendations based on disease stage.

Transarterial radioembolization (TARE) using resin or glass microspheres loaded with the radioactive isotope yttrium-90 is a targeted approach for the treatment of unresectable liver tumors. This method involves the direct delivery of a radiation source to the tumor through the hepatic artery, inducing necrosis in the tumor tissue and demonstrating notable immunomodulatory effects (Chew et al. 2019; Burnette and Weichselbaum 2015; Fernandez-Ros 2015). According to the current recommendations of the Barcelona Clinic Liver Cancer (BCLC) staging system, TARE is suggested for patients in BCLC stages of very early (0), early (A), or intermediate (B) (Reig et al. 2021). For patients in the advanced stage (C), TARE is not recommended because of insufficient evidence from clinical trials (EASL 2018, Vogel et al. 2021).

Despite the cautious stance in the current guidelines, TARE has proven to be effective in numerous patients. While prospective phase III trials did not establish TARE’s superiority over sorafenib, many individuals have exhibited a positive response to TARE (Chow et al. 2018; Vilgrain et al. 2017; Ricke et al. 2019). Additionally, recent meta-analyses of these trials have indicated the non-inferiority of TARE to sorafenib, coupled with greater tolerability of TARE (Venerito et al. 2020). Comparisons with transarterial chemoembolization (TACE), another standard locoregional treatment, did not reveal significant differences in terms of survival or adverse events (Pitton et al. 2015; Lobo et al. 2016; Tu et al. 2014).

Chronic inflammation, tissue remodelling, genetic alterations, and alterations in cellular signalling are the major factors responsible for the development of HCC (Katsanos et al. 2017; Fu et al. 2019; Michalopoulus 2010). Cytokines play a key role in this inflammatory process and promote tumor angiogenesis, tumor growth, metastasis, and invasion (Seidensticker et al. 2017). Interestingly, serum levels of interleukin (IL) -6 and − 8 have previously been shown to be associated with survival and liver function in a small population of patients with HCC treated with TARE (Morgan et al. 2008). However, the role of serum cytokines in predicting the outcome of HCC treatment has not been well established. The aim of our study was to investigate the value of serum cytokines associated with inflammation as potential biomarkers for better patient selection for TARE and to compare their predictive value with currently used liver function-based scores.

The selection of patients for TARE is not well established. Presently, assessing the success of TARE treatment often involves evaluating liver function using scores such as the albumin-bilirubin (ALBI) or Child-Pugh-Turcotte (CPT) scores (Demirtas et al. 2021; Johnson et al. 2015). Although the CPT score is widely used to grade liver function, it relies on non-standardized parameters such as encephalopathy and ascites. The ALBI score, introduced in 2014, offers an evidence-based and specifically designed scoring system to assess liver function in patients with hepatocellular carcinoma (HCC) patients (Johnson et al. 2015). The ALBI grade has demonstrated successful predictions of overall survival (OS) and recurrence-free survival (RFS), surpassing CPT in prognostic ability for patients with unresectable HCC undergoing TARE (Mohammadi et al. 2018; Antkowiak et al. 2019; Öcal et al. 2021). Given the lack of well-established criteria for selecting patients who will benefit from TARE using these scores and the overlap in indications for TARE, transarterial chemoembolization (TACE), and systemic treatment, there is a significant medical need to predict the response to TARE in the treatment of HCCs.

## Materials and methods

### Patients and serum samples

A total of 161 patients with HCC diagnosed by radiologic or histologic criteria who underwent Y^90^ radioembolization between 2010 and 2020 at our center were retrospectively assessed for inclusion in our study. Serum samples were available in a subset of 78 patients. Patients diagnosed with HCC at the intermediate stage (BCLC B) or advanced stage (BCLC C), with preserved liver function, and an Eastern Cooperative Oncology Group performance status ≤ 2 were eligible. A further prerequisite was the availability of paired serum samples taken before and at week 4 after TARE and stored at -20 °C. Written informed consent was obtained from all patients. This study was approved by the Ethics Committees of Medical Research of the University of Leipzig in accordance with the Declaration of Helsinki of 1975 (revision 2013) and the International Conference on Harmonization/Committee for Proprietary Medicinal Products “Good Clinical Practice” guidelines (Vote No. 132/23-ek).

Our findings were validated in an independent patient population participating in the prospective clinical trial SORAMIC, consisting of 83 patients treated with TARE in combination with the tyrosine kinase inhibitor sorafenib, which has been previously published (Öcal et al. 2021).

### Application of TARE

TARE was performed based on the recommendations of an interdisciplinary tumor board. According to our local treatment protocol, all patients underwent an evaluation angiography with embolization of hepato-gastric or hepato-mesenteric shunts if necessary and with application of a Tc-99 macroaggregated albumin scan (Tc-99 m-MAA) to evaluate the distribution of the therapeutic agent and to quantify lung shunting before TARE treatment. If the lung-shunt fraction was higher than 20%, TARE was not conducted. After the diagnostic angiography and Tc-99 m-MAA scintigraphy for treatment planning, a mean dose of 2.8 ± 1.3 [median 2.6 (range 0.5–6.1)] yttrium-90 glass or resin microspheres (TheraSphere, Boston Scientific, MA, USA; SIR-Spheres, Sirtex Medical Limited, MA, USA) was manually injected through a microcatheter. The distribution of microspheres in the tumor was recorded 24 h after treatment using single-photon emission computed tomography (SPECT).

### Quantification of serum cytokines

The concentrations of cytokines IL-1β, IL-6, IL-8, IL-10, IL-12p70, IL-17a, IL-18, IL-23, IL-33, interferon (IFN) -α2, INF-γ, tumor necrosis factor (TNF) -α, and monocyte chemoattractant protein-1 (MCP-1) were measured in serum samples using the LEGENDplex™ Human Inflammation Panel 1 (BioLegend, Fell, Germany) according to the manufacturer’s instructions.

### Statistical analysis

The following data were recorded for each patient from the time points before and 4 weeks after TARE: demographic and laboratory data, cancer characteristics, CTP score, model for end-stage liver disease (MELD) score, and ALBI score. The CTP score is based on total serum bilirubin and albumin and the international normalized ratio for prothrombin time (INR), as well as the quantification of the severity of ascites and hepatic encephalopathy from none to mild to severe (Child and Turcotte 1964). The patients were classified as previously described. The MELD sore included the serum levels of bilirubin and creatinine and INR, and is calculated according to the formula: MELD = 3.78 x ln (serum bilirubin [md/dL]) + 11.2 x ln (INR) + 9.57 x ln (serum creatinine [mg/dL]) + 6.43 (Kamath and Kim 2007). The ALBI score was calculated as previously described: ALBI = (log_10_ bilirubin [mmol/L] × 0.66) + (albumin [g/L] × (− 0.0852). ALBI classes were determined as follows: ALBI score ≤ − 2.60 (ALBI grade 1), − 2.60 to ≤ − 1.39 (ALBI grade 2), and ≥ − 1.39 (ALBI grade 3) (Johnson et al. 2015).

Statistical analyses of epidemiological associations were performed using the SPSS software (SPSS Inc., version 29.0, Chicago, IL, USA) and R software (version 4.2.3). Categorical variables are reported as frequencies and percentages. Values are presented as median and interquartile range unless otherwise specified. Fisher’s exact test was applied for categorical variables, and the Mann–Whitney U-test and Wilcoxon signed-rank test were used to compare quantitative variables. Spearman’s correlation coefficient was used to evaluate the relationship between cytokines, blood parameters, and liver function scores. The Kaplan–Meier method was used to estimates the 12-week and 24-week survival as well as the overall survival. The predictive value of biochemical parameters and cytokines was analyzed using receiver operating characteristic (ROC). The area under the ROC curve (AUROC), with values close to 1.0, indicated high diagnostic accuracy. The most accurate cut-off value was calculated using Youden’s index (Youden 1950). DeLong‘s test was applied to compare AUROCs of two or mor correlated predictors. Univariate and multivariate logistic regression analyses (inclusion models) were used to determine the association between different parameters and treatment response. Univariate and multivariate Cox regression models were used to assess the effects of confounding factors on 12-week, 24-week and overall survival. Odds ratios (OR), hazard ratios (HR), and 95% confidence intervals (CI) were calculated. Multivariate regression analysis was performed using *p* < 0.05 for inclusion and *p* > 0.1 for exclusion of parameters in the final model. All tests were two-sided, and statistical significance was set at p values < 0.05.

## Results

### Baseline characteristics

A total of 78 patients were included in the study. 79% (*n* = 62) of the patients were male, and the median age was 67 years (range 49–89; Table 1). The etiology of HCC was mainly alcoholic steatohepatitis (ASH) or mixed ASH/metabolic dysfunction-associated steatohepatitis (MASH), 30.8% and 12.8%, respectively). Liver cirrhosis was diagnosed in 83.3% of the patients. BCLC stages A, B, and C were evident in 3.8%, 65.4%, and 30.8% of the patients, respectively. The median MELD score was 8 (6–20), and the median ALBI score was − 2.68 (-3.56- -1.09). 53 (55%) patients were pretreated: 31 with TACE, 1 with TARE, 8 with systemic therapy, 2 with resection, and 1 with radiofrequency ablation.

**Table 1:** Baseline patients’ characteristics

Parameter	Patients (*n* = 78)
Male sex (%)	62 (79.5%)
Median age (years)†	67 (49–89)
Liver cirrhosis (%)	65 (83.3%)
CTP class (%)	
A	56 (71.8%)
B	9 (11.5%)
ALBI score†	-2.68 (-3.56 - -1.09)
ALBI grade*	
1	45 (57.7%)
2	30 (38.5%)
3	3 (3.8%)
MELD score†	8 (6–20)
HCC etiology (%)	
ASH	24 (30.8%)
ASH/MASH	10 (12.8%)
Cryptogenic	10 (12.8%)
Viral	5 (6.4%)
Others	29 (37.2%)
BCLC stage (%)	
A	3 (3.8%)
B	51 (65.4%)
C	24 (30.8%)
Tumor grading (%)	
G1	20 (25.6%)
G2	16 (20.5%)
G3	2 (2.6%)
Portal vein thrombosis (%)	12 (15.4%)
Macrovascular infiltration (%)	17 (21.8%)

†median (range), ALBI: albumin-bilirubin, ASH: alcoholic steatohepatitis, BCLC: Barcelona Clinic Liver Cancer, CTP: Child-Turcotte-Pugh, HCC: hepatocellular carcinoma, MASH: metabolic dysfunction-associated steatohepatitis, MELD: model for end-stage liver disease

The baseline patient characteristics from the SORAMIC trial are presented in Supplementary Table S1. The SORAMIC subgroup included significantly more males (92.8% vs. 79.5%, *p* = 0.020) and a higher number of patients with BCLC stage C (68.7% vs. 30.8%, *p* = 1.86 × 10^− 6^) than our study cohort.

A total of 47 (60%) patients in the study cohort and 73 (88%) patients in the SORAMIC trial died during the observation period (*p* = 5.95 × 10^− 5^). The median overall survival times were 36 weeks (4-436) and 44 weeks (7-167), respectively (shown in Supplementary Figure S1). In the study cohort, the overall survival rate after TARE was 5.2 ± 4.9%, 12-week survival rate was 89.5 ± 3.5%, and 24-week survival rate 70.9 ± 5.4%. In the SORAMIC trial, the overall survival rates were 0%, 97.6 ± 1.7% for 12-weeks and 79.5 ± 4.4% for 24 weeks.

### Biochemical parameters, liver function scores and cytokine levels before and after TARE

In the total population, there were significant changes in GGT levels (*p* = 5.44 × 10^− 7^), bilirubin levels (*p* = 4.80 × 10^− 6^), platelet count (*p* = 0.004), and MCP-1 levels (*p* = 0.008) between baseline and week 4 after TARE (Table 2). The mean ALBI score was also significantly higher after TARE (*p* = 1.30 × 10^− 6^).

**Table 2:** Blood parameters and serum cytokine concentrations before and at week 4 after TARE. Values are shown as median and range

Parameter	Before TARE		Week 4 after TARE		
	Median	Range	Median	Range	*p*-value
ALT [IU/L]	33	10–119	39	10–282	0.228
AP [IU/L]	146	47–653	139	49–886	0.568
**GGT [IU/L]**	203	35-1181	173	36–810	**5.44 × 10** ^**− 7**^
Leucocytes [10^9^/L]	5.9	2.3–15.5	5.5	1.3–2.4	0.741
Hemoglobin [gm/dL]	8.1	4.2–10.7	8.2	3.8–10.7	0.320
**Platelets [10** ^**9**^ **/L]**	132	40–512	129	47–380	**0.004**
Albumin [g/L]	39.1	26.1–47.1	38.9	22.1–47.0	0.255
**Bilirubin [µmol/L]**	12.9	3.8–59.5	17.1	4.0–67.0	**4.80 × 10** ^**− 6**^
Creatinine [µmol/L]	78	45–211	77	41–285	0.254
**ALBI score**	-2.68	-3.56–1.09	-2.51	-3.61–1.15	**1.30 × 10** ^**− 6**^
AFP [ng/mL]	40.2	1.8-60500	80.8	3.0-60500	0.165
IL-1β [pg/mL]	9.28	1.50-548.31	8.81	1.50-1234.16	0.700
IL-6 [pg/mL]	14.75	2.26-130.94	16.29	2.04–152.60	0.194
IL-8 [pg/mL]	44.62	2.61-6237.74	56.87	26.10-1934.50	0.521
IL-10 [pg/mL]	5.47	2.00-193.00	7.46	2.00-113.76	0.149
IL-12p70 [pg/mL]	3.08	2.00-90.47	3.08	2.00-46.69	0.964
IL-17a [pg/mL]	1.40	0.50-17.45	3.62	0.50–66.40	0.180
IL-18 [pg/mL]	198.10	7.18-1123.44	198.51	1.33-1303.44	0.872
IL-23 [pg/mL]	7.44	1.80-157.30	10.19	1.80-117.86	0.413
IL-33 [pg/mL]	72.32	4.40-338.93	24.31	4.40-396.49	0.571
IFN-α2 [pg/mL]	10.25	2.10-58.74	5.28	2.10-47.53	0.276
IFN-γ [pg/mL]	12.25	1.30-76.01	6.84	1.30-80.98	0.143
TNF-α [pg/mL]	48.15	0.90-338.16	16.09	0.90-194.71	0.468
**MCP-1 [pg/mL]**	395.18	36.03-3793.18	347.29	29.61-1572.21	**0.008**

AFP: alpha-fetoprotein, ALBI: albumin-bilirubin, ALT: alanine aminotransferase, AP: alkaline phosphatase, GGT: gamma-glutamyl transpeptitase, IFN: interferon, IL: interleukin, MCP-1: monocyte chemoattractant protein-1, TARE: transarterial radioembolization, TNF: tumor necrosis factor

**Fig. 1:**
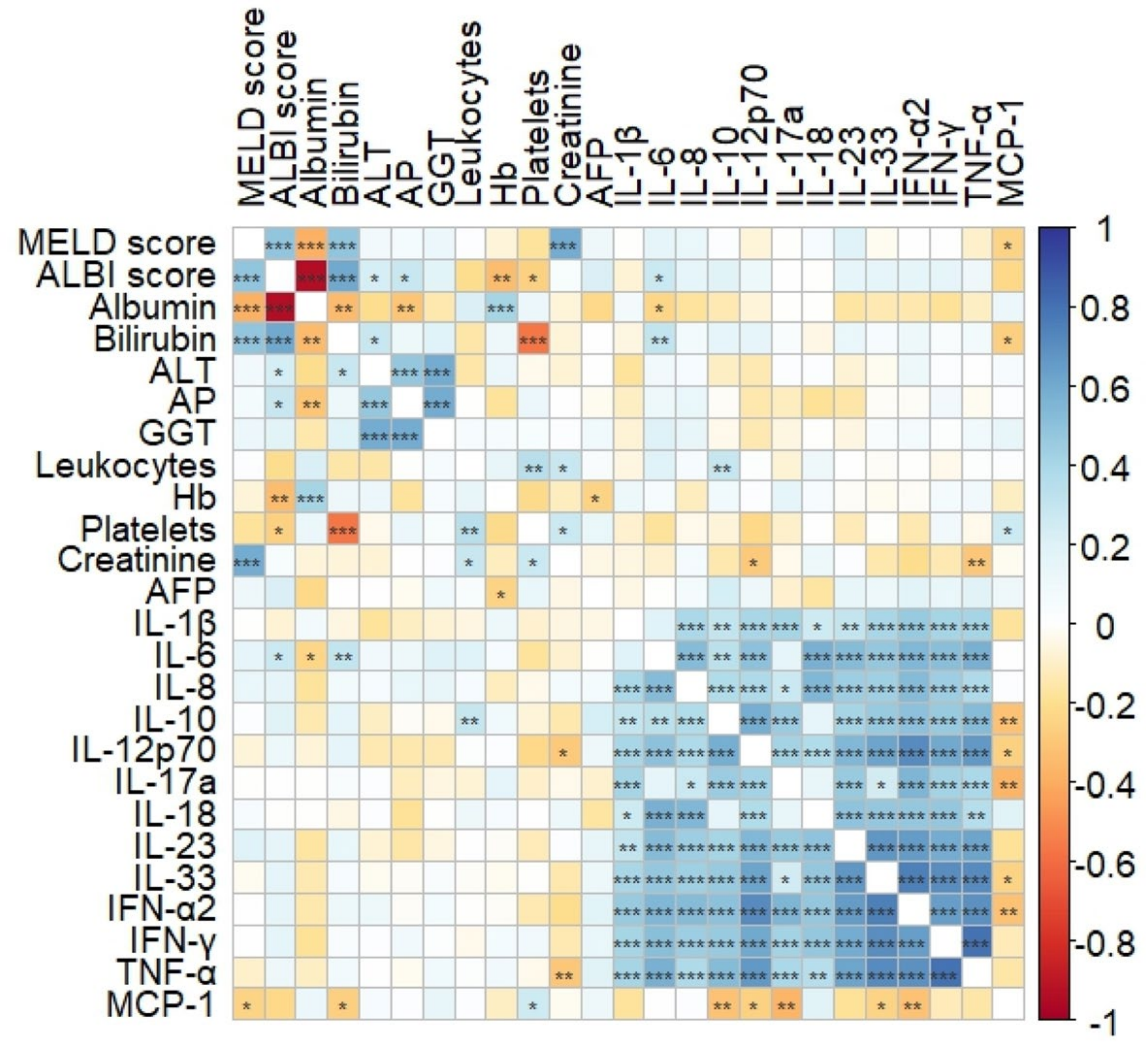
Correlation matrix of biochemical parameters, cytokines and liver function scores in patients with HCC before TARE. The intensity of the colors indicates the Spearman’s correlation coefficient between two parameters. The asterisks display the p-values with * *p* < 0.05, ** *p* < 0.01 and *** *p* < 0.001. Significance was tested using a Pearson correlation test and level of significance was set at *p* < 0.05. ALBI: albumin-bilirubin, ALT: alanine aminotransferase, GGT: gamma-glutamyl transpeptitase, Hb: hemoglobin, IFN: interferon, IFN-g: interferon gamma, IL: interleukin, MCP-1: monocyte chemoattractant protein-1, MELD: model for end-stage liver disease, TNF: tumor necrosis factor

Before TARE, pro-inflammatory cytokines such as IL-12p70, IL-17a, IL23, and IL-33, as well as the anti-inflammatory cytokine IL-10, showed intermediate to high correlation with the interferons IFN-α2, IFN-γ, and TNF-α, which are known to be associated with apoptosis, cell proliferation, and cell differentiation. There was a weak correlation of IL-6 with ALBI score (*r* = 0.276, *p* = 0.016), albumin (*r*=-0.230, *p* = 0.045) and bilirubin (*r* = 0.306, *p* = 0.007). IL-8 showed an intermediate correlation with IL-6 (*p* = 0.524, *p* = 1.21 × 10^− 6^), IL-1β (*r* = 0.392, *p* = 0.0005) and IL-10 (*p* = 0.379, *p* = 0.001) (shown in Fig. 1).

### Association of biochemical parameters, liver function scores and cytokine levels with 12-week and 24-week survival after TARE

Baseline IL-8 levels (309.80 vs. 40.50 pg/mL, *p* = 7.40 × 10^− 4^ and MELD score (10 vs. 6, *p* = 0.017) were significantly higher in patients who died 12 weeks after TARE than in those who survived (shown in Supplementary Table S2).

**Fig. 2:**
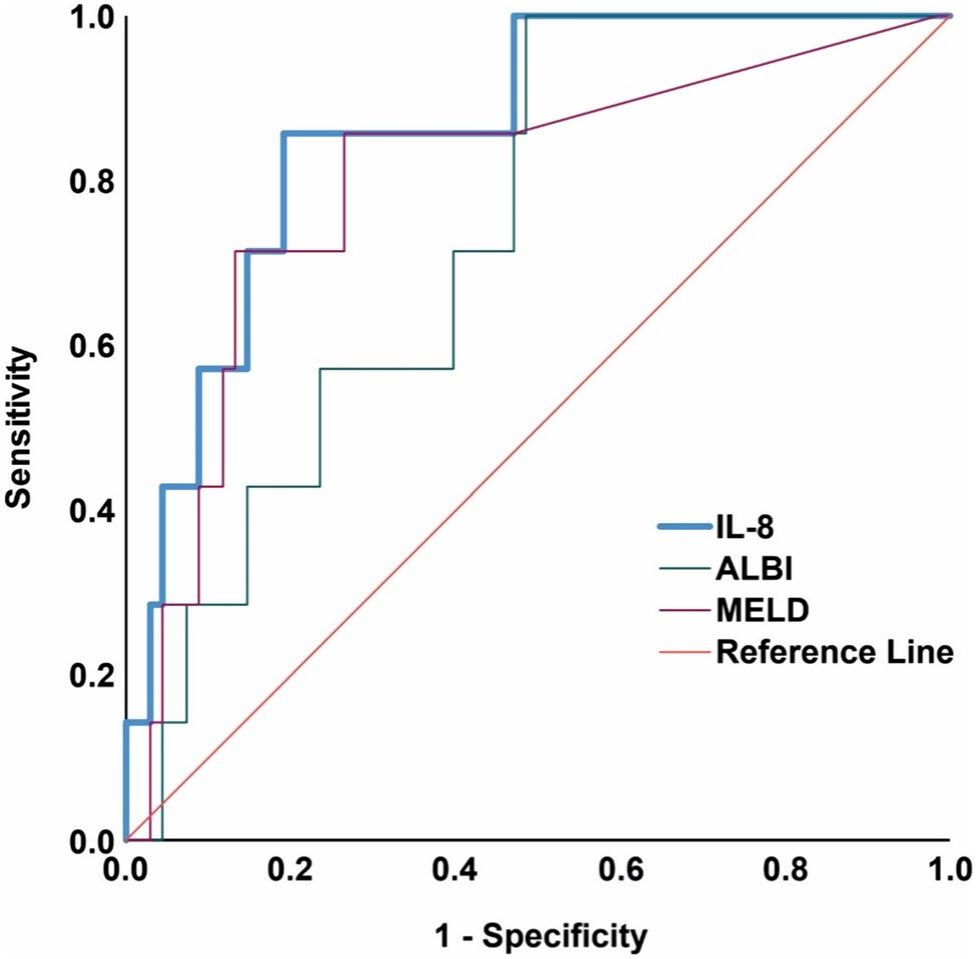
Receiver operating characteristics (ROC) curve of the association of IL-8, ALBI and MELD score with 12-week survival after TARE

ROC analysis was performed to determine how accurately IL-8 and the liver function scores ALBI and MELD could discriminate between patients with and without survival ≥ 12 weeks after TARE (shown in Fig. 2). The best performance was observed for baseline IL-8, with an area under the receiver operating characteristic curve (AUROC) of 0.861 (*p* = 0.002), followed by the MELD score with AUROC = 0.799 (*p* = 0.009) and ALBI score with AUROC = 0.735 (*p* = 0.041). However, the IL-8 AUROC was not significantly different from the MELD score AUROC (Z = 0.505, *p* = 0.614) and ALBI score AUROC (Z = 1.499, *p* = 0.134). The cut-off value for the 12-week survival discrimination for IL-8 was 190 pg/mL, with a sensitivity of 85.7% and specificity of 81.2%. The positive predictive value (PPV) and negative predictive value (NPV) for identifying patients with 12-week survival were 98.2% and 31.6%, respectively.

Median baseline IL-1β levels (5.28 vs. 10.67 pg/mL, *p* = 0.021) and IL-17a levels (0.54 vs. 2.06, *p* = 0.031) were significantly lower in patients who died 24 weeks after TARE compared to those who survived (Supplementary Table S3).

### Multivariate analysis of factors associated with 12-week and 24-week survival after TARE

Univariate Cox regression analysis showed a significant association between an IL-8 cut-off of 190 pg/mL and 12-week (*p* = 0.006) and 24-week survival (*p* = 0.039). The MELD score (*p* = 0.025) was also associated with 12-week survival. Alterations in the ALBI score, representing liver function, showed no significant impact on 12-week (*p* = 0.147) and 24-week survival (*p* = 0.421). In the multivariate Cox regression analysis, the IL-8 cut-off remained independently associated with 12-week and 24-week survival (Table 3).

**Table 3:** Univariate and multivariate analyses of factors associated with 12-week and 24-week survival after TARE

Parameter	12-week survival				24-week survival				
	Univariate analysis		Multivariate analysis		Univariate analysis			Multivariate analysis	
	HR (95% CI)	*p* value	HR (95% CI)	*p* value	HR (95% CI)	*p* value	HR (95% CI)		*p* value
IL-8 cut-off > 190 pg/mL	19.01 (2.29-157.89)	**0.006**	**15.82 (1.90-131.91)**	**0.011**	2.57 (2.05–6.31)	**0.039**	**3.04 (1.06–8.73)**		**0.039**
Male sex	0.42 (0.10–1.77)	0.238			0.73 (0.27–1.98)	0.530			
Age [years]	0.99 (0.91–1.08)	0.833			0.99 (0.94–1.04)	0.751			
Liver cirrhosis	1.47 (0.18–11.97)	0.717			4.17 (0.56–31.08)	0.164			
CTP class B vs. A	2.60 (0.51–5.60)	0.253			1.29 (0.38–4.39)	0.688			
BCLC C vs. B	0.68 (0.14–3.53)	0.633			1.54 (0.65–3.66)	0.326			
Macrovascular infiltration	0.48 (0.06–3.93)	0.497			0.90 (0.33–2.47)	0.326			
Portal vein thrombosis	0.81 (0.10–6.58)	0.843			0.54 (0.13–2.32)	0.406			
AFP [ng/mL]	1.00 (1.00–1.00)	0.106			1.00 (1.00–1.00)	**0.036**	**1.00 (1.00–1.00)**		**0.026**
Albumin [g/L]	0.91 (0.80–1.03)	0.147			0.96 (0.89–1.05)	0.404			
Bilirubin [µmol/L]	1.04 (0.99–1.09)	0.103			1.02 (0.98–1.06)	0.391			
ALBI score	2.31 (0.74–7.18)	0.147			1.38 (0.63–3.06)	0.421			
MELD score	1.18 (1.02–1.36)	**0.025**	1.18 (1.00-1.40)	0.056	1.04 (0.92–1.18)	0.507			

AFP: alpha-fetoprotein, ALBI: albumin-bilirubin, BCLC: Barcelona Clinic Liver Cancer score, CI: confidence interval, CTP: Child-Turcotte-Pugh, HR: hazard ratio, IL: interleukin, MELD: model for end-stage liver disease

### Association of the IL-8 cut-off of 190 pg/mL with 12-week and 24-week survival after TARE in the test cohort and in the SORAMIC validation cohort

Patients with baseline IL-8 levels above the cut-off of 190 pg/mL had a significantly lower 12-week survival rate after TARE than patients with lower IL-8 levels (66.9 ± 11.1% vs. 98.2%±1.7%, *p* = 9.27 × 10^− 5^). Patients’ characteristics of both groups are shown in Supplementary Table S4.

**Fig. 3:**
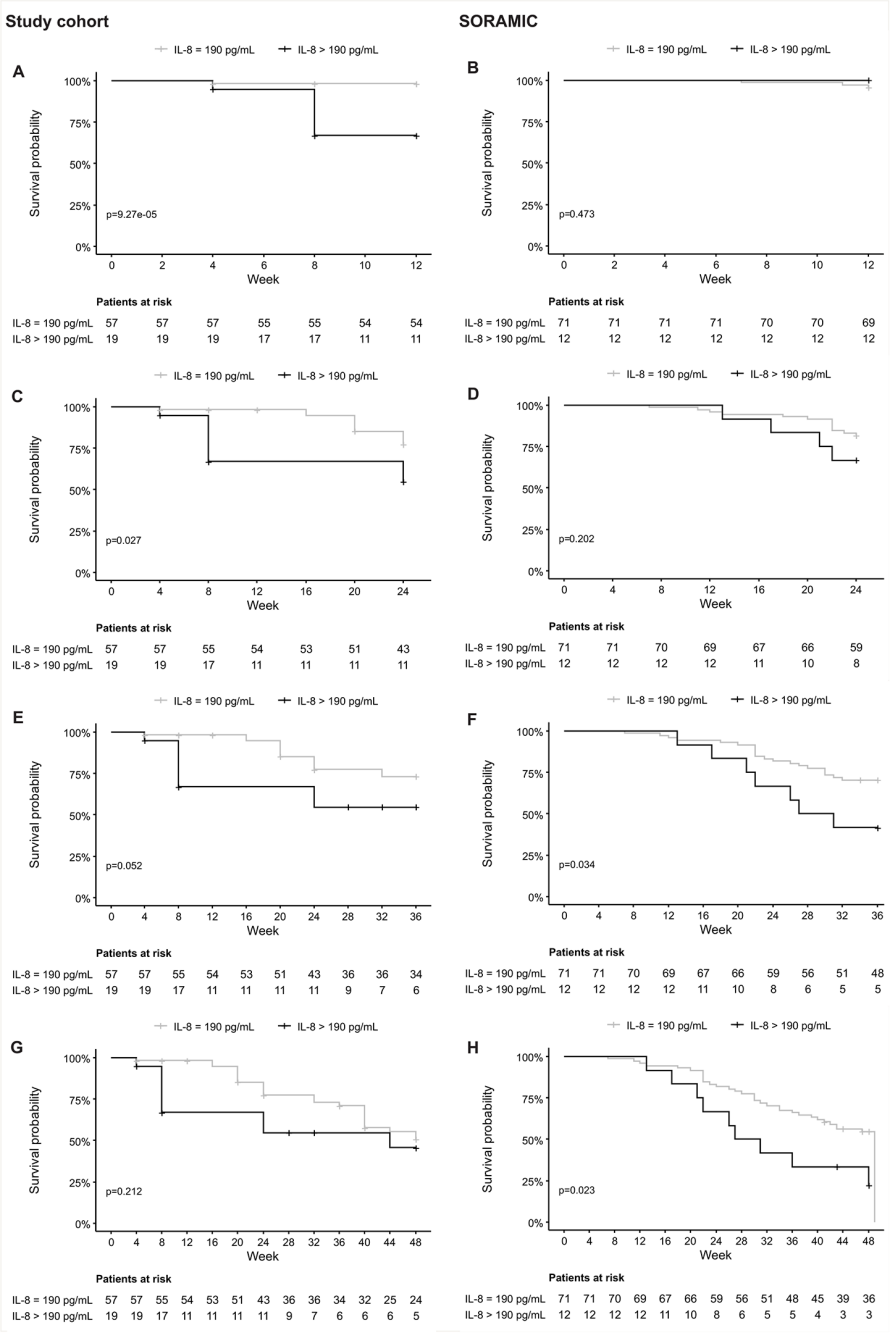
Kaplan-Meier curves are showing survival of the study cohort at 12 weeks (**A**), 24 weeks (**B**) and 36 weeks (**C**) and of the patients in the SORAMIC trial at 24 weeks (**D**), 36 weeks (**E**) and 48 weeks (**F**) after TARE according to the baseline interleukin (IL)-8 cut-off of 190 pg/mL

This association was also found for the 24-week survival rates (54.7 ± 11.9% vs. 77.8 ± 5.8%, *p* = 0.027). Although statistical significance was not achieved, differences in survival rates were observed during the 36-week survival period. Specifically, the survival rate was 54.7 ± 11.9% for individuals with IL-8 > 190 pg/mL, in contrast to 73.0 ± 6.2% for those with IL-8 ≤ 190 pg/mL (*p* = 0.052) (shown in Fig. 3A-C). Additionally, patients with IL-8 levels > 190 pg/mL before TARE experienced significantly shorter overall survival, with a median survival time of 24 weeks (range: 4-196) compared to 40 weeks (range: 4-436) in patients with IL-8 levels ≤ 190 pg/mL (*p* = 0.039).

In the TARE + sorafenib subgroup of the SORAMIC cohort (*n* = 83), the IL-8 cut-off of 190 pg/mL was able to distinguish patients at high risk of death from patients at high likelihood of survival at week 36 (41.7%±14.2 vs. 70.4%±5.4%, *p* = 0.034) and at week 48 (22.2%±12.8 vs. 54.7 ± 5.9%, *p* = 0.023) after TARE (shown in Fig. 3D-F). Additionally, the median overall survival time significantly differed between patients with IL-8 > 190 pg/mL and patients with IL-8 ≤ 190 pg/mL, with median survival times of 29 weeks (13–114) and 49 weeks (7-167), respectively (*p* = 0.026).

## Discussion

In the dynamic landscape of current treatment options for HCC, which is characterized by a range of locoregional therapies and a growing propensity for systemic interventions, identifying suitable candidates for locoregional approaches, such as TARE, in clinical practice is challenging. This challenge is particularly pronounced in patients with intermediate- or advanced-stage HCC as systemic treatments are increasingly available for these patients. Biomarkers for the selection of patients with a good prognosis after TARE represent a high medical need but are not currently available.

Within a substantial and carefully characterized population of HCC patients undergoing TARE, our study revealed a remarkable correlation between pre-TARE interleukin-8 (IL-8) levels and key indicators of liver function and necro-inflammation. Our results emphasize that setting an IL-8 cutoff value of < 190 pg/mL at the time of TARE allows the identification of patients with an increased probability of survival at 12 and 24 weeks after TARE, with a sensitivity and specificity of 86% and 81%, respectively.

Remarkably, this identified IL-8 threshold of < 190 pg/mL also proved effective in identifying patients who survived for up to 48 weeks in an independent validation cohort treated with TARE plus sorafenib, as observed in the prospective and randomized SORAMIC trial. In contrast to IL-8, the levels of other cytokines, including IL-1β, IL-6, IL-10, IL-12p70, IL-17a, IL-18, IL-23, IL-33, interferon (IFN)-α2, INF-γ, TNF-α, and MCP-1, were unrelated to survival after TARE (Supplementary Table S2).

IL-8 (also known as CXCL8) is a cytokine that mediates its actions by binding to one of the two chemokine receptors, CXCR1 or CXCR2. IL-8 is physiologically produced by monocytes, endothelial cells, and various epithelial cells, and is an angiogenesis mediator and pro-inflammatory chemotactic factor for neutrophils that enhances tumor cell growth and promotes angiogenesis (Sannamed et al. 2014; Koch et al. 1992). IL-8 has previously been shown to reflect HCC tumor burden and correlate with tumor stage in patients with HCC (Sannamed et al. 2014). Consistent with our findings, IL-8 levels are associated with treatment response and survival after TACE in patients with primary and metastatic liver tumors (Lossen et al. 2018). Another prospective study evaluating cytokines in HCC or metastatic disease has shown that a cut-off value of 6.53 pg/mL for IL-6 and 60.8 pg/mL for IL-8 was associated with overall survival after TARE (Öcal et al. 2021). The correlation between IL-8 serum levels and survival after TARE, which we were able to show in our study, could be related to the role of IL-8 as a potent mediator of inflammation. However, other properties of IL-8, such as its role in the tumor microenvironment, could also contribute to this finding. IL-8 is often associated with tumor progression and angiogenesis (Alfaro et al. 2017). It attracts immune cells, promotes inflammation, and contributes to the invasive properties of cancer cells. This seems particularly conclusive, as the IL-8 signaling pathway has previously been associated with promoting cancer cell survival and proliferation, as well as stimulating cancer cell migration and invasion (Alfaro et al. 2017). IL-8 is also a potent trigger of angiogenesis, a property that could contribute to the identification of patients with poor survival in our study. These properties were less pronounced or absent in the other cytokines that were examined in our study (i.e., IL-1β, IL-6, IL-10, IL-12p70, IL-17a, IL-18, IL-23, and IL-33) and might contribute to their lack of association with survival in patients with tumors in our study. Interestingly, IL-8 has been described to have a strong influence on the prognosis of patients undergoing cancer immunotherapy (Bakouny and Coueiri 2020). Therefore, we believe that the immunological interaction of TARE and IL-8 needs to be investigated in future studies, as well as the association of IL-8 with immunotherapies, which have become the standard first-line treatment for HCC (EASL 2018, Vogel et al. 2021).

In addition, we found that among others IL-6 and IL-10 levels correlated with IL-8 levels, which may reflect the inflammatory microenvironment of the tumor (shown in Fig. 1), the latter also known for its inhibitory effect on the immunosurveillance (Shakiba et al. 2018). Interestingly, MCP-1 levels decreased significantly 4 weeks after TARE. Furthermore, MCP-1 has been shown to be a predictive marker for tumor response in patients undergoing TACE (You et al. 2021). In our patient population, IL-8 showed an intermediate to high correlation with ALT and bilirubin levels, which further indicated a potential association with the inflammatory tumor environment in these patients. However, serum cytokines other than IL-8 were not associated with survival in our population, a finding that differs from that of previous reports. For example, Öcal et al. (2021) reported a significant association between IL-6 and overall survival, even in multivariate analysis, while Carpizo et al. (2014) reported 22 patients (7 with HCC) after TARE with survival of more than 6 months who had lower baseline IL-8 levels but no correlation with IL-6. The reason for the differences between these studies and our study may be due to the different characteristics of the study populations and differences in treatment.

In our patient population, IL-8 levels performed better in identifying patients with short survival (< 12 weeks) after TARE than liver function-related MELD and CTP scores (shown in Fig. 2). We found that an IL-8 threshold of > 190 pg/mL was useful for identifying patients with poor survival (shown in Fig. 3). Our study thus confirms previous results that suggest a correlation between cytokines and TARE treatment outcomes in patients with HCC, although our cut-off for IL-8 levels differs (Seidensticker et al. 2017). Importantly, we were able to validate our results in an independent patient cohort from the prospective SORAMIC trial (shown in Fig. 3). While distinctions in survival were not immediately evident in SORAMIC patients, they became apparent at 36 and 48 weeks post-TARE. This delay in observation is likely attributable to the low mortality rate within the initial months following TARE. Notably, patients with extended survival periods could still be identified by using a threshold value of 190 pg/mL. Considering the different selections of this patient cohort and the fact that these patients had received TARE in combination with sorafenib, we interpret these results as a clear confirmation that IL-8 levels are a potential predictive marker for survival in patients undergoing TARE.

The retrospective nature is a limitation of our study. The differences in the findings of both the training and the validation cohort could be related to real-world (training) and prospectively selected patients (SORAMIC). A notable strength of our study is the validation of our findings within an independent cohort from a prospectively collected clinical trial. In conclusion, we have shown that the levels of IL-8 have high potential to become a clinical tool for the selection of patients who benefit from TARE. Future studies on the potential of IL-8 in predicting the response to different HCC therapies and on the specific role of IL-8 in HCC pathogenesis are needed to further define its potential role as a decision aid for the management of patients with HCC.

## Electronic supplementary material

Below is the link to the electronic supplementary material.

Supplementary Material 1

Supplementary Material 2

## Data Availability

No datasets were generated or analysed during the current study.

## Abbreviations

AFP: Alpha-fetoprotein
ALBI: Albumin-bilirubin
ALT: Alanine aminotransferase
AP: Alkaline phosphatase
ASH: Alcoholic steatohepatitis
AUROC: Area under the ROC curve
BCLC: Barcelona clinic liver cancer
CI: Confidence interval
CT: Computed tomography
CTP: Child-Turcotte-Pugh
GGT: Gamma-glutamyl transpeptitase
Hb: Hemoglobin
HCC: Hepatocellular carcinoma
HR: Hazard ratio
IFN: Interferon
IL: Interleukin
INR: International normalized ratio
MCP-1: Monocyte chemoattractant protein-1
MELD: Model for end-stage liver disease
mRECIST: Response Evaluation Criteria in Solid Tumors
MASH: Metabolic dysfunction-associated steatohepatitis
MRI: Magnetic resonance imaging
NPV: Negative predicting value
OR: Odds ratio
OS: Overall survival
PPV: Positive predictive value
RFS: Recurrence-free survival
ROC: Receiver operating characteristic
TACE: Transarterial chemoembolization
TARE: Transarterial radioembolization
Tc-99m-MAA: Tc-99 macro aggregated albumin
TNF: Tumor necrosis factor

## Declarations

### Registry and Registration No. of the study/trail

N/A.

### Animal studies

N/A.

### Research involving recombinant DNA

N/A.
