# Supplementary material for: Impact of IL-8 on survival after TARE in HCC: a comprehensive investigation and external validation from the SORAMIC trial
Source: J Cancer Res Clin Oncol. 2024 Nov 6;150(11):486. doi: 10.1007/s00432-024-05947-4 (PMC11541297; doi:10.1007/s00432-024-05947-4)
Supplement: Supplementary file 1 — Supplementary Material 1 [file 432_2024_5947_MOESM1_ESM.pdf]

Supplementary Material

Interleukin-8 and survival after transarterial radioembolization in hepatocellular carcinoma: a comprehensive study and external validation from the SORAMIC trial

Aaron Schindler*[1,5], Janett Fischer*[1,5], Anne-Bettina Beeskow[2,5], Thomas Lincke [3,5], Sebastian Ebel [2,5], Daniel Seehofer[4,5], Timm Denecke [2,5], Rhea Veelken[1], Osama Sabri[3,5], Osman Öcal[6], Max Seidensticker[6], Thomas Berg[1,5], Florian van Bömmel[1,5]

*****shared first authorship

Author affiliations:

1) Division of Hepatology, Department of Medicine II, Leipzig University Medical Center, , Germany
2) Department of Diagnostic and Interventional Radiology, Leipzig University Medical Center, Leipzig, Germany
3) Department of Nuclear Medicine, Leipzig University Medical Center, 04103 Leipzig, Germany.
4) Department of Visceral, Thoracic and Vascular Surgery, Leipzig University Medical Center, Germany.
5) University Liver Tumor Center (ULTC), Leipzig University Medical Center, Leipzig, Germany
6) Division of Hepatology, Department of Medicine II, Leipzig University Medical Center, Laboratory for Clinical and Experimental Hepatology, Leipzig, Germany
7) Department of Radiology, University Hospital, LMU Munich, Munich, Germany

Corresponding Author:
Prof. Dr. Florian van Bömmel, MD
Division of Hepatology
Department of Medicine II
Leipzig University Medical Center
Liebigstrasse 20
04103 Leipzig
Phone: +49 (0)3419712330
Fax: +49 (0)3419712339
E-Mail: florian.vanboemmel@medizin.uni-leipzig.de

Supplementary Table S1: Baseline patients' characteristics of the SORAMIC study cohort treated with transarterial embolization in combination with sorafenib

Parameter	Patients (n=83)
Male sex (%)	77 (92.8%)
Median age (years)†	66 (44-81)
Liver cirrhosis (%)	71 (85.5%)
CTP class (%)	
A	64 (90.1%)
B	6 (8.5%)
ALBI score†	-2.66 (-3.39 - -1.56)
ALBI grade†	
1	42 (51.2%)
2	40 (48.8%)
BCLC stage (%)	
A	0 (0%)
B	26 (31.3%)
C	57 (68.7%)
IL-8 [pg/mL]†	53.60 (0.67-2591.35)
Albumin [g/L]†	39.1 (29.0-48.3)
Bilirubin [µmol/L] †	12.0 (3.0-42.8)

†median (range), ALBI: albumin-bilirubin, BCLC: Barcelona Clinic Liver Cancer, CTP: Child-Turcotte-Pugh, IL: interleukin, MELD: model for end-stage liver disease

Supplementary Table S2: Association of baseline biochemical parameters, liver function scores and serum cytokine levels with survival beyond 12 weeks. Values are shown as median and range.

Parameter	Survival < 12 weeks after TARE (n=8)		Survival ≥ 12 weeks after TARE (n=70)		p-value
	**Median**	**Range**	**Median**	**Range**	
ALT [IU/L]	44	16-119	33	10-101	0.349
AP [IU/L]	157	101-301	141	47-653	0.472
GGT [IU/L]	311	35-799	201	47-1181	0.568
Leucocytes [10^9/L]	6.2	3.6-10.5	5.9	2.3-15.5	0.370
Hemoglobin [gm/dL]	7.7	4.2-9.0	8.1	5.6-10.7	0.187
Platelets [10^9/L]	144	52-219	129	40-512	0.695
Albumin [g/L]	37.2	26.5-44.8	39.3	26.1-47.1	0.235
Bilirubin [µmol/L]	14.4	6.2-59.5	12.8	3.8-43.8	0.444
Creatinine [µmol/L]	46	50-191	77	45-211	0.051
ALBI score	-2.49	-3.33--1.44	-2.72	-3.56--1.09	0.143
**MELD Score**	**10**	**6-17**	**6**	**6-20**	**0.017**
IL-1β [pg/mL]	5.77	2.42-377.00	9.28	1.50-548.31	0.536
IL-6 [pg/mL]	37.17	3.40-87.14	14.53	2.26-130.90	0.077
**IL-8 [pg/mL]**	**309.80**	**44.49-6237.74**	**40.5**	**2.61-2034.06**	**7.40×10^{-4}**
IL-10 [pg/mL]	13.31	2.00-106.38	5.02	2.00-193.00	0.206
IL-12p70 [pg/mL]	3.08	2.00-90.47	3.08	2.00-51.88	0.753
IL-17a [pg/mL]	1.48	0.50-3.80	1.36	0.50-17.45	0.718
IL-18 [pg/mL]	280.47	93.40-1123.44	190.98	7.18-932.69	0.176
IL-23 [pg/mL]	11.61	3.07-129.35	5.78	1.80-157.30	0.273
IL-33 [pg/mL]	35.29	4.40-338.93	26.94	4.40-309.97	0.781
IFN-α2 [pg/mL]	12.48	2.10-58.74	3.91	2.10-45.58	0.067
IFN-γ [pg/mL]	6.23	1.30-19.89	6.78	1.59-76.01	0.688
TNF-α [pg/mL]	9.47	0.90-64.01	1161	0.90-338.16	0.989
MCP-1 [pg/mL]	236.93	73.59-3793.18	401.00	36.03-1191.08	0.752

ALBI: albumin-bilirubin, ALT: alanine aminotransferase, AST: aspartate aminotransferase, AP: alkaline phosphatase, GGT: gamma-glutamyl transpeptitase, IFN: interferon, IL: interleukin, MCP-1:monocyte chemoattractant protein-1, MELD: model for end-stage liver disease, TNF: tumor necrosis factor

Supplementary Table S3: Baseline biochemical parameters, liver function scores and serum cytokine levels with 24-week survival. Values are shown as median and range.

Parameter	Survival < 24 weeks after TARE (n=21)		Survival ≥ 24 weeks after TARE (n=57)		
	**Median**	**Range**	**Median**	**Range**	**p-value**
ALT [IU/L]	38	16-119	33	10-101	0.229
AP [IU/L]	152	81-653	143	47-452	0.358
GGT [IU/L]	272	35-1181	201	47-694	0.403
Leucocytes [10^9/L]	6.2	3.6-15.5	5.8	2.3-9.5	0.306
Hemoglobin [gm/dL]	7.8	4.2-9.3	8.2	5.6-10.7	0.112
Platelets [10^9/L]	146	40-302	127	51-512	0.435
Albumin [g/L]	38.4	27.2-47.1	39.2	26.1-47.0	0.288
Bilirubin [µmol/L]	12.5	6.2-59.5	13.2	3.8-43.8	0.425
Creatinine [µmol/L]	83	45-191	77	45-211	0.338
ALBI score	-2.64	-3.43--1.39	-2.72	-3.56--1.09	0.591
MELD Score	6	6-17	6	6-20	0.430
**IL-1β [pg/mL]**	**5.28**	**2.42-377.00**	**10.67**	**1.50-548.31**	**0.021**
IL-6 [pg/mL]	16.46	3.40-130.94	14.75	2.26-100.66	0.248
IL-8 [pg/mL]	142.36	2.62-6237.74	41.16	2.61-1185.60	0.289
IL-10 [pg/mL]	7.89	2.00-106.38	4.75	2.00-193.00	0.323
IL-12p70 [pg/mL]	3.60	2.00-90.47	3.08	2.00-51.88	0.756
**IL-17a [pg/mL]**	**0.54**	**0.50-16.97**	**2.06**	**0.50-17.45**	**0.031**
IL-18 [pg/mL]	253.07	7.18-1123.44	189.76	17.73-932.69	0.478
IL-23 [pg/mL]	8.75	1.80-157.30	5.30	1.80-146.97	0.923
IL-33 [pg/mL]	20.73	4.40-338.93	30.49	4.40-309.97	0.798
IFN-α2 [pg/mL]	7.71	2.10-58.74	4.11	2.10-45.58	0.561
IFN-γ [pg/mL]	8.15	1.30-31.63	6.44	1.59-76.01	0.736
TNF-α [pg/mL]	14.90	0.90-82.12	11.34	0.90-338.16	0.705
MCP-1 [pg/mL]	403.65	73.59-3793.18	395.18	36.03-1191.08	0.884

ALBI: albumin-bilirubin, ALT: alanine aminotransferase, AST: aspartate aminotransferase, AP: alkaline phosphatase, GGT: gamma-glutamyl transpeptitase, IFN: interferon, IL: interleukin, MCP-1:monocyte chemoattractant protein-1, MELD: model for end-stage liver disease, TNF: tumor necrosis factor

Supplementary TableS4: Patients' characteristics of the IL-8 ≤ 190 pg/ml and IL-8 >190pg/ml groups.

**Parameter**	**IL-8 ≤ 190 pg/ml (n=57)**	**IL-8 >190pg/ml (n=19)**	**p-value**
Male sex (%)	45 (78.9%)	16 (84.2%)	0.748
Median age (years)†	67 (49-89)	67 (52-79)	0.297
Liver cirrhosis (%)	45 (78.9%)	19 (100%)	0.031
CTP class (%)			
A	40 (88.9%)	15 (78.9%)	
B	5 (11.1%)	4 (21.1%)	0.432
ALBI score†	-2.76 (-3.56 - -1.09)	-2.57 (-3.43 - -1.27)	0.091
ALBI grade*			
1	35 (61.4%)	8 (42.8%)	
2	20 (35.1%)	10 (52.6%)	0.302
3	2 (3.5%)	1 (5.3%)	
MELD score†	6 (6-20)	6 (6-17)	0.186
BCLC stage (%)			
A	0 (0%)	3 (15.8%)	
B	39 (68.4%)	11 (57.9%)	0.022
C	18 (31.6%)	5 (26.3%)	
Portal vein thrombosis (%)	7 (12.3%)	5 (26.3%)	0.161
Macrovascular infiltration (%)	13 (22.8%)	4 (21.1%)	1.000
ALT [IU/L]†	0.55 (0.17-1.67)	0.65 (0.31-1.99)	0.198
AP [IU/L]†	2.35 (0.78-10.89)	2.61 (1.76-5.02)	0.350
GGT [IU/L]†	3.35 (0.86-14.95)	4.01 (0.78-19.68)	0.238
Leucocytes [10^9/L]†	6.3 (2.3-15.5)	5.6 (3.5-8.8)	0.180
Hemoglobin [gm/dL]†	8.1 (6.3-10.7)	7.7 (4.2-9.7)	0.208
Platelets [10^9/L]†	138 (40-512)	123 (52-284)	0.389
Albumin [g/L]†	40.2 (27.2-47.0)	38.4 (26.1-47.1)	0.069
Bilirubin [µmol/L]†	12.8 (3.8-43.8)	14.7 (7.8-59.5)	0.330
Creatinine [µmol/L]†	78 (45-211)	77 (50-191)	0.419
AFP [ng/ml]†	28.9 [1.8-60500)	348.5 (2.3-38590)	0.678

†median (range), AFP: alpha-fetoprotein, ALBI: albumin-bilirubin, ALT: alanine aminotransferase, AP: alkaline phosphatase, BCLC: Barcelona Clinic Liver Cancer, CTP: Child-Turcotte-Pugh, GGT: gamma-glutamyl transpeptitase, IL-8: interleukin 8, MELD: model for end-stage liver disease

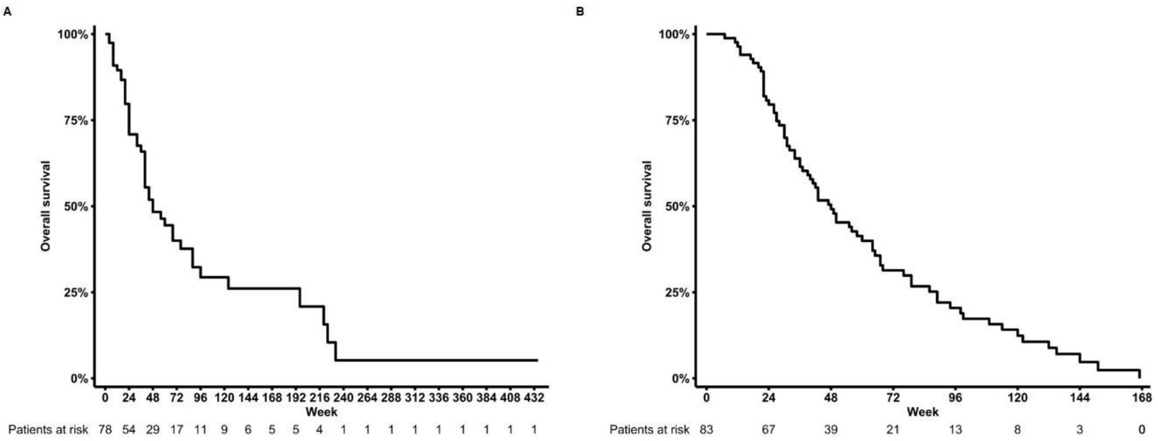

Supplementary Figure S1. Kaplan-Meier curves are showing the overall survival in patients after TARE of the study cohort (A) and SORAMIC trial (B).
