# Supplementary material for: Impact of IL-8 on survival after TARE in HCC: a comprehensive investigation and external validation from the SORAMIC trial
Source: J Cancer Res Clin Oncol. 2024 Nov 6;150(11):486. doi: 10.1007/s00432-024-05947-4 (PMC11541297; doi:10.1007/s00432-024-05947-4)
Supplement: Supplementary file 2 — Supplementary Material 2 [file 432_2024_5947_MOESM2_ESM.docx]

**Supplementary Material**

**Interleukin-8 and survival after transarterial radioembolization in hepatocellular carcinoma: a comprehensive study and external validation from the SORAMIC trial**

Aaron Schindler*^1,5^, Janett Fischer*^1,5^, Anne-Bettina Beeskow^2,5^, Thomas Lincke ^3,5^, Sebastian Ebel ^2,5^, Daniel Seehofer^4,5^, Timm Denecke ^2,5^, Rhea Veelken^1^, Osama Sabri^3,5^, Osman Öcal^6^, Max Seidensticker^6^, Thomas Berg^1,5^, Florian van Bömmel^1,5^

*****shared first authorship

Author affiliations:

1. Division of Hepatology, Department of Medicine II, Leipzig University Medical Center, , Germany
2. Department of Diagnostic and Interventional Radiology, Leipzig University Medical Center, Leipzig, Germany
3. Department of Nuclear Medicine, Leipzig University Medical Center, 04103 Leipzig, Germany.
4. Department of Visceral, Thoracic and Vascular Surgery, Leipzig University Medical Center, Germany.
5. University Liver Tumor Center (ULTC), Leipzig University Medical Center, Leipzig, Germany
6. Division of Hepatology, Department of Medicine II, Leipzig University Medical Center, Laboratory for Clinical and Experimental Hepatology, Leipzig, Germany
7. Department of Radiology, University Hospital, LMU Munich, Munich, Germany

**Corresponding Author:**

Prof. Dr. Florian van Bömmel, MD

Division of Hepatology

Department of Medicine II

Leipzig University Medical Center

Liebigstrasse 20

04103 Leipzig

Phone: +49 (0)3419712330

Fax: +49 (0)3419712339

E-Mail: [florian.vanboemmel@medizin.uni-leipzig.de](mailto:florian.vanboemmel@medizin.uni-leipzig.de)

**Supplementary Table S1:** Baseline patients’ characteristics of the SORAMIC study cohort treated with transarterial embolization in combination with sorafenib

| **Parameter** | **Patients (n=83)** |
| --- | --- |
| Male sex (%) | 77 (92.8%) |
| Median age (years)^†^ | 66 (44-81) |
| Liver cirrhosis (%) | 71 (85.5%) |
| CTP class (%) |  |
| A | 64 (90.1%) |
| B | 6 (8.5%) |
| ALBI score^†^ | -2.66 (-3.39 - -1.56) |
| ALBI grade^†^ |  |
| 1 | 42 (51.2%) |
| 2 | 40 (48.8%) |
| BCLC stage (%) |  |
| A | 0 (0%) |
| B | 26 (31.3%) |
| C | 57 (68.7%) |
| IL-8 [pg/mL]^†^ | 53.60 (0.67-2591.35) |
| Albumin [g/L]^†^ | 39.1 (29.0-48.3) |
| Bilirubin [µmol/L] ^†^ | 12.0 (3.0-42.8) |
| ^†^median (range), ALBI: albumin-bilirubin, BCLC: Barcelona Clinic Liver Cancer, CTP: Child-Turcotte-Pugh, IL: interleukin, MELD: model for end-stage liver disease | |

**Supplementary Table S2:** Association of baseline biochemical parameters, liver function scores and serum cytokine levels with survival beyond 12 weeks. Values are shown as median and range.

| **Parameter** | **Survival < 12 weeks after TARE (n=8)** | | **Survival ≥ 12 weeks after TARE (n=70)** | |  |
| --- | --- | --- | --- | --- | --- |
|  | **Median** | **Range** | **Median** | **Range** | **p-value** |
| ALT [IU/L] | 44 | 16-119 | 33 | 10-101 | 0.349 |
| AP [IU/L] | 157 | 101-301 | 141 | 47-653 | 0.472 |
| GGT [IU/L] | 311 | 35-799 | 201 | 47-1181 | 0.568 |
| Leucocytes [10^9^/L] | 6.2 | 3.6-10.5 | 5.9 | 2.3-15.5 | 0.370 |
| Hemoglobin [gm/dL] | 7.7 | 4.2-9.0 | 8.1 | 5.6-10.7 | 0.187 |
| Platelets [10^9^/L] | 144 | 52-219 | 129 | 40-512 | 0.695 |
| Albumin [g/L] | 37.2 | 26.5-44.8 | 39.3 | 26.1-47.1 | 0.235 |
| Bilirubin [µmol/L] | 14.4 | 6.2-59.5 | 12.8 | 3.8-43.8 | 0.444 |
| Creatinine [µmol/L] | 46 | 50-191 | 77 | 45-211 | 0.051 |
| ALBI score | -2.49 | -3.33--1.44 | -2.72 | -3.56--1.09 | 0.143 |
| **MELD Score** | **10** | **6-17** | **6** | **6-20** | **0.017** |
| IL-1β [pg/mL] | 5.77 | 2.42-377.00 | 9.28 | 1.50-548.31 | 0.536 |
| IL-6 [pg/mL] | 37.17 | 3.40-87.14 | 14.53 | 2.26-130.90 | 0.077 |
| **IL-8 [pg/mL]** | **309.80** | **44.49-6237.74** | **40.5** | **2.61-2034.06** | **7.40x10^-4^** |
| IL-10 [pg/mL] | 13.31 | 2.00-106.38 | 5.02 | 2.00-193.00 | 0.206 |
| IL-12p70 [pg/mL] | 3.08 | 2.00-90.47 | 3.08 | 2.00-51.88 | 0.753 |
| IL-17a [pg/mL] | 1.48 | 0.50-3.80 | 1.36 | 0.50-17.45 | 0.718 |
| IL-18 [pg/mL] | 280.47 | 93.40-1123.44 | 190.98 | 7.18-932.69 | 0.176 |
| IL-23 [pg/mL] | 11.61 | 3.07-129.35 | 5.78 | 1.80-157.30 | 0.273 |
| IL-33 [pg/mL] | 35.29 | 4.40-338.93 | 26.94 | 4.40-309.97 | 0.781 |
| IFN-α2 [pg/mL] | 12.48 | 2.10-58.74 | 3.91 | 2.10-45.58 | 0.067 |
| IFN-γ [pg/mL] | 6.23 | 1.30-19.89 | 6.78 | 1.59-76.01 | 0.688 |
| TNF-α [pg/mL] | 9.47 | 0.90-64.01 | 1161 | 0.90-338.16 | 0.989 |
| MCP-1 [pg/mL] | 236.93 | 73.59-3793.18 | 401.00 | 36.03-1191.08 | 0.752 |
| ALBI: albumin-bilirubin, ALT: alanine aminotransferase, AST: aspartate aminotransferase, AP: alkaline phosphatase, GGT: gamma-glutamyl transpeptitase, IFN: interferon, IL: interleukin, MCP-1:monocyte chemoattractant protein-1, MELD: model for end-stage liver disease, TNF: tumor necrosis factor | | | | | |

**Supplementary Table S3:** Baseline biochemical parameters, liver function scores and serum cytokine levels with 24-week survival. Values are shown as median and range.

| **Parameter** | **Survival < 24 weeks after TARE (n=21)** | | **Survival ≥ 24 weeks after TARE (n=57)** | |  |
| --- | --- | --- | --- | --- | --- |
|  | **Median** | **Range** | **Median** | **Range** | **p-value** |
| ALT [IU/L] | 38 | 16-119 | 33 | 10-101 | 0.229 |
| AP [IU/L] | 152 | 81-653 | 143 | 47-452 | 0.358 |
| GGT [IU/L] | 272 | 35-1181 | 201 | 47-694 | 0.403 |
| Leucocytes [10^9^/L] | 6.2 | 3.6-15.5 | 5.8 | 2.3-9.5 | 0.306 |
| Hemoglobin [gm/dL] | 7.8 | 4.2-9.3 | 8.2 | 5.6-10.7 | 0.112 |
| Platelets [10^9^/L] | 146 | 40-302 | 127 | 51-512 | 0.435 |
| Albumin [g/L] | 38.4 | 27.2-47.1 | 39.2 | 26.1-47.0 | 0.288 |
| Bilirubin [µmol/L] | 12.5 | 6.2-59.5 | 13.2 | 3.8-43.8 | 0.425 |
| Creatinine [µmol/L] | 83 | 45-191 | 77 | 45-211 | 0.338 |
| ALBI score | -2.64 | -3.43--1.39 | -2.72 | -3.56--1.09 | 0.591 |
| MELD Score | 6 | 6-17 | 6 | 6-20 | 0.430 |
| **IL-1β [pg/mL]** | **5.28** | **2.42-377.00** | **10.67** | **1.50-548.31** | **0.021** |
| IL-6 [pg/mL] | 16.46 | 3.40-130.94 | 14.75 | 2.26-100.66 | 0.248 |
| IL-8 [pg/mL] | 142.36 | 2.62-6237.74 | 41.16 | 2.61-1185.60 | 0.289 |
| IL-10 [pg/mL] | 7.89 | 2.00-106.38 | 4.75 | 2.00-193.00 | 0.323 |
| IL-12p70 [pg/mL] | 3.60 | 2.00-90.47 | 3.08 | 2.00-51.88 | 0.756 |
| **IL-17a [pg/mL]** | **0.54** | **0.50-16.97** | **2.06** | **0.50-17.45** | **0.031** |
| IL-18 [pg/mL] | 253.07 | 7.18-1123.44 | 189.76 | 17.73-932.69 | 0.478 |
| IL-23 [pg/mL] | 8.75 | 1.80-157.30 | 5.30 | 1.80-146.97 | 0.923 |
| IL-33 [pg/mL] | 20.73 | 4.40-338.93 | 30.49 | 4.40-309.97 | 0.798 |
| IFN-α2 [pg/mL] | 7.71 | 2.10-58.74 | 4.11 | 2.10-45.58 | 0.561 |
| IFN-γ [pg/mL] | 8.15 | 1.30-31.63 | 6.44 | 1.59-76.01 | 0.736 |
| TNF-α [pg/mL] | 14.90 | 0.90-82.12 | 11.34 | 0.90-338.16 | 0.705 |
| MCP-1 [pg/mL] | 403.65 | 73.59-3793.18 | 395.18 | 36.03-1191.08 | 0.884 |
| ALBI: albumin-bilirubin, ALT: alanine aminotransferase, AST: aspartate aminotransferase, AP: alkaline phosphatase, GGT: gamma-glutamyl transpeptitase, IFN: interferon, IL: interleukin, MCP-1:monocyte chemoattractant protein-1, MELD: model for end-stage liver disease, TNF: tumor necrosis factor | | | | | |

**Supplementary TableS4:** Patients’ characteristics of the IL-8 ≤ 190 pg/ml and IL-8 >190pg/ml groups.

| **Parameter** | **IL-8 ≤ 190 pg/ml (n=57)** | **IL-8 >190pg/ml (n=19)** | **p-value** |
| --- | --- | --- | --- |
| Male sex (%) | 45 (78.9%) | 16 (84.2%) | 0.748 |
| Median age (years)† | 67 (49-89) | 67 (52-79) | 0.297 |
| Liver cirrhosis (%) | 45 (78.9%) | 19 (100%) | 0.031 |
| CTP class (%) |  |  |  |
| A | 40 (88.9%) | 15 (78.9%) |  |
| B | 5 (11.1%) | 4 (21.1%) | 0.432 |
| ALBI score† | -2.76 (-3.56 - -1.09) | -2.57 (-3.43 - -1.27) | 0.091 |
| ALBI grade* |  |  |  |
| 1 | 35 (61.4%) | 8 (42.8%) |  |
| 2 | 20 (35.1%) | 10 (52.6%) | 0.302 |
| 3 | 2 (3.5%) | 1 (5.3%) |  |
| MELD score† | 6 (6-20) | 6 (6-17) | 0.186 |
| BCLC stage (%) |  |  |  |
| A | 0 (0%) | 3 (15.8%) |  |
| B | 39 (68.4%) | 11 (57.9%) | 0.022 |
| C | 18 (31.6%) | 5 (26.3%) |  |
| Portal vein thrombosis (%) | 7 (12.3%) | 5 (26.3%) | 0.161 |
| Macrovascular infiltration (%) | 13 (22.8%) | 4 (21.1%) | 1.000 |
| ALT [IU/L]† | 0.55 (0.17-1.67) | 0.65 (0.31-1.99) | 0.198 |
| AP [IU/L]† | 2.35 (0.78-10.89) | 2.61 (1.76-5.02) | 0.350 |
| GGT [IU/L]† | 3.35 (0.86-14.95) | 4.01 (0.78-19.68) | 0.238 |
| Leucocytes [10^9^/L]† | 6.3 (2.3-15.5) | 5.6 (3.5-8.8) | 0.180 |
| Hemoglobin [gm/dL]† | 8.1 (6.3-10.7) | 7.7 (4.2-9.7) | 0.208 |
| Platelets [10^9^/L]† | 138 (40-512) | 123 (52-284) | 0.389 |
| Albumin [g/L]† | 40.2 (27.2-47.0) | 38.4 (26.1-47.1) | 0.069 |
| Bilirubin [µmol/L]† | 12.8 (3.8-43.8) | 14.7 (7.8-59.5) | 0.330 |
| Creatinine [µmol/L]† | 78 (45-211) | 77 (50-191) | 0.419 |
| AFP [ng/ml]† | 28.9 [1.8-60500) | 348.5 (2.3-38590) | 0.678 |
| †median (range), AFP: alpha-fetoprotein, ALBI: albumin-bilirubin, ALT: alanine aminotransferase, AP: alkaline phosphatase, BCLC: Barcelona Clinic Liver Cancer, CTP: Child-Turcotte-Pugh, GGT: gamma-glutamyl transpeptitase, IL-8: interleukin 8, MELD: model for end-stage liver disease | | | |

**
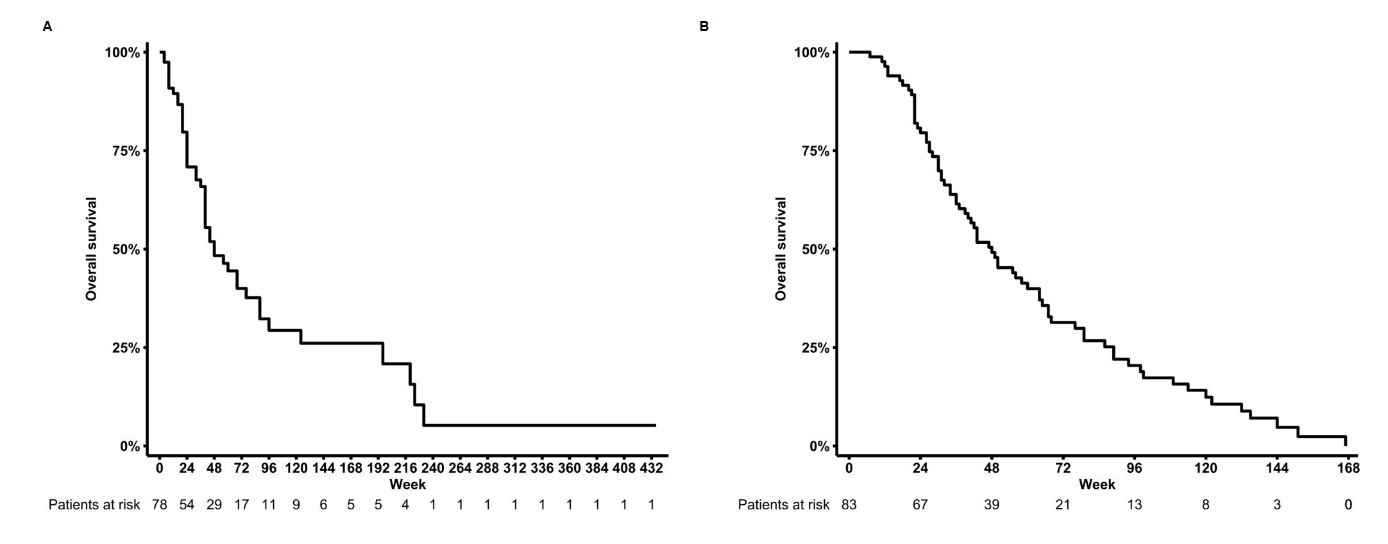
**

**Supplementary Figure S1.** Kaplan-Meier curves are showing the overall survival in patients after TARE of the study cohort (A) and SORAMIC trial (B).
